# Detection of plant oil addition to cheese by synchronous fluorescence spectroscopy

**DOI:** 10.1007/s13594-015-0218-5

**Published:** 2015-03-15

**Authors:** Anna Dankowska, Maria Małecka, Wojciech Kowalewski

**Affiliations:** 1 Faculty of Commodity Science, Poznań University of Economics, Poznań, Poland; 2 Faculty of Mathematics and Computer Science, Adam Mickiewicz University, Poznań, Poland

**Keywords:** Cheese, Food adulteration, Milk fat, Food quality, Synchronous fluorescence spectroscopy, Multivariate data analysis

## Abstract

The fraudulent addition of plant oils during the manufacturing of hard cheeses is a real issue for the dairy industry. Considering the importance of monitoring adulterations of genuine cheeses, the potential of fluorescence spectroscopy for the detection of cheese adulteration with plant oils was investigated. Synchronous fluorescence spectra were collected within the range of 240 to 700 nm with different wavelength intervals. The lowest detection limits of adulteration, 3.0 and 4.4%, respectively, were observed for the application of wavelength intervals of 60 and 80 nm. Multiple linear regression models were used to calculate the level of adulteration, with the lowest root mean square error of prediction and root mean square error of cross validation equalling 1.5 and 1.8%, respectively, for the measurement acquired at the wavelength interval of 60 nm. Lower classification errors were obtained for the successive projections algorithm-linear discriminant analysis (SPA–LDA) rather than for the principal component analysis (PCA)–LDA method. The lowest classification error rates equalled 3.8% (∆*λ* = 10 and 30 nm) and 0.0% (∆*λ* = 60 nm) for the PCA–LDA and SPA–LDA classification methods, respectively. The applied technique is useful for detecting the addition of plant fat to hard cheese.

## Introduction

Milk, cheese, and other dairy products are consumed worldwide and have great commercial importance within the food industry. Cheese is made from milk; therefore, the only fat it contains is milk fat. Cheese-like products are obtained by partial or total substitution of milk fat by significantly cheaper plant oils. Milk fat is one of the most expensive commodity fats on the market; therefore, adulteration of cheese is practiced for economic purposes and detection of foreign fat in milk fat is a real issue. The most common adulterants of cheese are palm, coconut, corn, and cotton oils (Alejewicz et al. 2011).

Various instrumental methods have been proposed to establish the authenticity of cheese and to detect the level of its adulteration. Among the methods focused on detecting foreign fat in milk products are the PCR-based techniques (Plath et al. 1997), capillary and gel electrophoresis (Cartoni et al. 1999; Veloso et al. 2004; Guerreiro et al. 2013), HPLC (Veloso et al. 2004), immunochemical methods (Hurley et al. 2006; Pizzano et al. 2011; Rodríguez et al. 2010), GC (Kim et al. 2014), front-face fluorescence spectroscopy (Hammami et al. 2013; Karoui et al. 2007) and fluorescence spectroscopy (Ntakatsane et al. 2013).

Synchronous fluorescence spectroscopy is an alternative technique that is quick and avoids all sample preparation steps except for dilution and therefore is simpler, less costly and quicker than other most widely used techniques. In the synchronous fluorescence technique, both excitation and emission monochromators are scanned simultaneously, with a constant wavelength interval maintained between excitation and emission wavelengths. As opposed to other conventional fluorescence techniques, synchronous fluorescence spectroscopy makes it possible to simplify spectra, to reduce spectral overlaps and to achieve greater selectivity (Patra and Mishra 2002).

The application of chemometric methods, e.g. multiple linear regression (MLR) or linear discriminant analysis (LDA), to spectrophotometric data requires selecting spectral variables for building well-fitted models. It is a challenge to select the proper analytical wavelengths from a spectrum. The successive projections algorithm (SPA) is an approach suitable for selecting effective wavelength variables from the spectra. SPA performs simple operations in a vector space to determine a subset of variables with minimal collinearity. SPA is described in detail by Araújo et al. (2001) and Soares et al. (2013). First, in an orthogonal sub-space, the vector of higher projection is selected and becomes the new starting vector. The choice of the orthogonal sub-space at each iteration is made in order to minimise the collinearity of variables. SPA has been compared to the genetic algorithm, which is a popular method for variable selection in multivariate calibration, and the results proved to be in favour of SPA (Araújo et al. 2011). It was found to be less sensitive to instrumental noise than the genetic algorithm. Moreover, the SPA-MLR models proved to be comparable to or even better than the full-spectrum partial last squares (PLS) or principal component regression (PCR) models for UV–Vis (Araújo et al. 2011) as well as the low-resolution plasma spectra analysis (Galvão et al. 2001). SPA has been used for variable selection in studies aimed at the classification of coffees {UV–Vis} (Polari Souto et al. 2010) and edible seed oils {UV–Vis and synchronous fluorescence spectroscopy} (Dankowska et al. 2013a), as well as olive oil {synchronous fluorescence spectroscopy} (Dankowska et al. 2013b).

The aim of the study is to evaluate the potential of synchronous fluorescence spectroscopy followed by chemometric analysis (successive projection algorithm (SPA) combined with multiple linear regression (MLR) and linear discriminant analysis (LDA)) for the detection of cheese adulteration with plant oils. This evaluation was performed on the basis of established errors of prediction of adulteration, percentage of misclassified samples and calculation of limits of detection of plant oil addition into cheese fat. To the best of the author’s knowledge, it was the first attempt at using synchronous fluorescence for the detection of cheese adulteration and at choosing wavelengths from the synchronous fluorescence spectra of cheese fats using the successive projections algorithm (SPA).

## Materials and methods

### Chemical reagents and samples

Samples of 21 cheeses and five cheese-like products were purchased at a local market in Poznań, Poland. The only fat content in the cheese-like products was plant fat. All samples were stored under refrigerated conditions until analysis.

Fat extraction from cheese and cheese-like samples was performed according to the Folch et al. (1957) method using a mix of chloroform and methanol (2:1, *v*/*v*). Then, 50 g of cheese samples was mixed with 200 mL of the chloroform–methanol mixture and homogenised for 10 min. The homogenised mixture was then filtered through filter paper and the first 150 mL of filtered extraction mixture was collected in the cylinder. Next, 30 mL of 0.74% KCl aqueous solution was added. The alcohol–water phase was removed, and the chloroform phase containing lipids was evaporated under a vacuum in a rotary evaporator.

To develop an analytical model, a set of fats extracted from the cheeses (*n* = 21) was melted at 60 °C and blended together to make a cheese fat stock. Simultaneously, the fat extracted from the cheese-like products (*n* = 6) was mixed to prepare two cheese-like product stocks (each adulterant stock contained fat extracted from three cheese-like products). The model adulterant mixtures were constructed by spiking the cheese fat stocks with two cheese-like product stocks at levels ranging from 10 to 90% at 10% intervals (*w*/*w*), resulting in 18 mixtures.

Two series of experimental mixtures were prepared, which together with fat extracted from cheese and cheese-like products yielded 48 {27 (cheese and cheese-like product fats) + 21 (mixtures and fat stocks)} samples to be analysed in duplicate. All reagents used were of analytical grade. Synchronous fluorescence spectra were collected for each extracted fat sample to develop a classification model.

### Synchronous fluorescence spectra measurement

Fluorescence spectra were obtained on a Fluorolog 3-11 spectrofluorometer, Spex-Jobin Yvon S.A. with a xenon lamp as a source of excitation. Excitation and emission slit widths were 2 nm. The acquisition interval and integration time were maintained at 1 nm and 0.1 s, respectively (Sikorska et al. 2005). The spectra were fully corrected for the wavelength response of the system. Right-angle geometry was used for oil samples diluted in *n*-hexane (1% *v*/*v*) in a 10-mm fused quartz cuvette. The synchronous fluorescence spectra were acquired by simultaneously scanning the excitation and emission monochromators within the excitation wavelength range of 240 to 700 nm, with constant wavelength distances ∆*λ* between them. Four spectra were collected for each sample, for wavelength intervals of 10, 30, 60 and 80 nm. Fluorescence intensities were plotted as a function of the excitation wavelength.

### Statistical analysis

The successive projections algorithm (SPA) was coded in C++. The purpose of SPA is to select wavelengths with minimally redundant information content in order to solve the collinearity problem. SPA employs operations in a vector space to obtain variables with the smallest collinearity. The initial variable and the number of variables can be given as input information or can be determined on the basis of the smallest root mean squared error of prediction in the validation set of the calibration model (Araújo et al. 2011). In this study, while the number of variables to be selected was given as input information while the initial variable was chosen to minimise the root mean square error of calibration (RMSEC) obtained for MLR analysis.

Multiple linear regression (MLR) and principal component analysis (PCA) as well as linear discriminant analysis (LDA) and the calculation of limits of detection (LOD) and multivariate limits of detection (MLD) were performed using Statistica 10.0 (StatSoft Inc., Tulsa, USA). Multiple linear regression and linear regression, in turn, permitted the calculation of the detection limits of adulteration of cheese fat with plant fat. The leave-one-out cross validation method was applied to evaluate the MLR models. For the MLR models, root mean square errors of calibration and validation were calculated. Detection limits of foreign oils in cheese fat were calculated to establish the effectiveness of this method. LDA permitted the discrimination of genuine cheeses from cheese-like products.

## Results and discussion

### Synchronous fluorescence spectra of cheese and cheese-like product fat

**Fig. 1:**
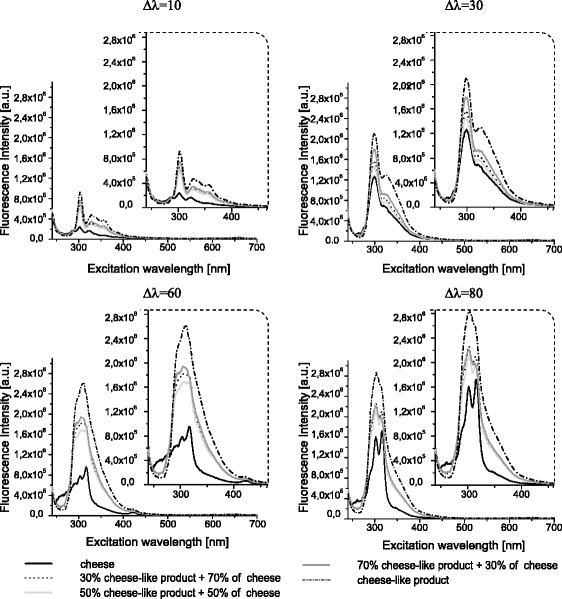
Synchronous fluorescence spectra of cheese fat, cheese-like product fat and their mixtures at different wavelength intervals

Fluorescence spectra intensities obtained for the fat extracted from cheese and cheese-like products and their mixtures were plotted as a function of the excitation wavelength (Fig. 1). Cheese fat and plant fat exhibit differences in fluorescence intensities, which makes it possible to distinguish between cheese and plant fats on the basis of their fluorescence spectra. The diversification of synchronous fluorescence spectra of cheese fat and plant fat by using different wavelength intervals is shown in Fig. 1. Fluorescence spectra intensities acquired for cheese and plant fats depend on the content of tocopherols, tocotrienols and chlorophylls (Ntakatsane et al. 2013). The spectra presented in Fig. 1 clearly indicate the potential of synchronous fluorescence for the discrimination between cheese fat and plant fat. In order not to make Fig. 1 illegible, the results for only three mixtures of cheese fat with plant fat were presented. Most fluorescent intensities decreased along the wavelength interval.

### Principal component analysis

**Fig. 2:**
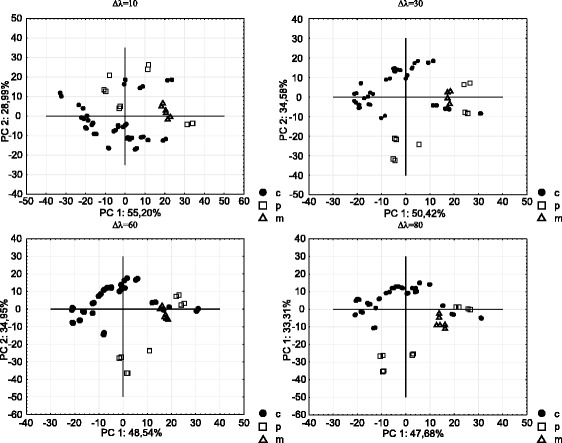
PCA plots of synchronous fluorescence intensities acquired at different wavelength intervals (*filled circles* indicate cheese fat, *open squares* indicate cheese-like product fat and *open triangles* indicate mixtures of cheese fat with cheese-like product fat)

Figure 2 presents the PCA plots (PC1 × PC2) of synchronous fluorescence spectra obtained for all cheese fats and cheese-like product fat samples and their mixtures in the proportion 1:1 (50% of adulteration) at different wavelength intervals. Similar PCA visual distinction ability was observed for all wavelength intervals. Cheese and cheese-like product samples formed two clusters regardless of the wavelength interval used. However, clusters partially overlapped each other. The cluster formed by the mixtures of cheese and cheese-like products overlaps both clusters formed by cheese samples and cheese-like product samples. The PCA analysis of cheeses and cheese-like product spectra indicates the potential of synchronous fluorescence for discrimination between both groups of products.

### Selection of wavelengths using the successive projection algorithm

In this experiment, the successive projections algorithm was applied to retain the most informative wavelengths from the spectra for further chemometric analysis. In this experiment, the number of wavelengths to be selected (5) was given as input information for all wavelength intervals (∆*λ* = 10, 30, 60 and 80 nm). It was possible to compare the separation abilities of the data obtained at different wavelength intervals, because the number of wavelengths to be selected was determined. The first wavelength was chosen by minimising the root mean square error of calibration (RMSEC) of the multiple linear regression model (MLR) for the prediction of percentage addition.

**Fig. 3:**
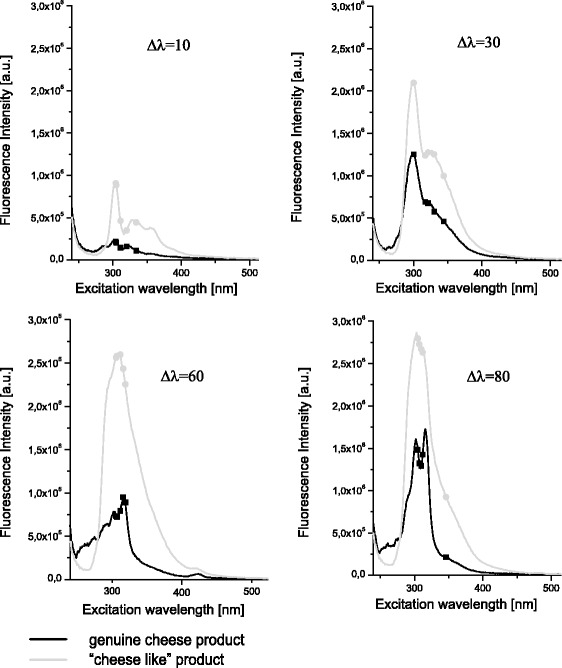
Synchronous fluorescence spectra of cheese fat and cheese-like product fat at different wavelength intervals. Wavelengths selected with the use of the successive projection algorithm are marked by *squares* and *circles*

As a result of the successive projections algorithm (SPA) analysis of the synchronous fluorescence spectra, five wavelengths for each of the wavelength intervals were chosen for further analysis: 305, 304, 320, 311 and 334 nm (∆*λ* = 10 nm); 300, 330, 321, 317 and 344 (∆*λ* = 30 nm); 300, 312, 307, 316 and 306 (∆*λ* = 60 nm) and 307, 312, 310, 305 and 346 (∆*λ* = 80 nm). The wavelengths selected by the SPA for each of the wavelength intervals are indicated with squares and circles in Fig. 3. Furthermore, five variables were selected from among 20 previously selected wavelengths to build a global model: 321 (Δ*λ* = 30 nm), 316 (Δ*λ* = 60 nm), 307 (Δ*λ* = 60 nm), 319 (Δ*λ* = 60 nm) and 312 (Δ*λ* = 80 nm). The fluorescence intensities obtained for the measurements at each wavelength interval (10, 30, 60 and 80 nm) at wavelengths chosen previously by the SPA algorithm were again given as input information for the SPA to select wavelengths for the global model analysis. The rule for choosing the first wavelength was the same as for the individual model.

### Synchronous fluorescence intensities versus the addition of an adulterant

A multiple regression analysis was applied to the previously selected fluorescence intensities of the experimental mixtures of cheese and cheese-like fats and their mixtures (Table 1). Multiple regression analysis models were built separately for the data acquired at each wavelength interval (10, 30, 60 and 80 nm). Apart from individual models for each of the wavelength intervals, a global model was built. The root mean square errors of calibration (RMSEC) and the root mean square errors of cross validation (RMSECV) calculated by means of the leave-one-out method (Table 1) made it possible to asses and confirm the prediction ability of the models. The RMSEC and RMSECV values calculated for the global model are lower than for the individual models. The lowest RMSEC and RMSECV for individual models equalled 1.5 and 1.8, respectively, and were obtained at the wavelength interval of 60 nm. The RMSEC and RMSECV for the global model equalled 0.7 and 0.8, respectively. The predicted concentration of plant oil in butter fat may be calculated on the basis of the equations presented in Table 2. The results are comparable to the ones obtained by Rodríguez et al. (2010). The RMSECV of the PLS calibration models obtained for the detection of cheese adulteration in their study varied from 1.05 to 6.99%. The results obtained for models built to detect the adulteration of other milk products are similar. Heussen et al. (2007) used the near infrared spectra to predict the butter content of spreads with the RMSEC ranging from 4.3 to 8.2% (*w*/*w*), depending on the details of the model used. Raman spectroscopy enables the determination of butter adulteration with margarine, with the RMSECV at 7.8 and 8.8 for the PCR and PLS models, respectively (Uysal et al. 2013).

**Table 1:** Statistical characteristics of multiple regression models calculated for the data obtained at different wavelength intervals applied in synchronous fluorescence measurements and for the global model

Parameter	Δ*λ* = 10	Δ*λ* = 30	Δ*λ* = 60	Δ*λ* = 80	Global model
RMSEC [%]	1.8	1.7	*1.5*	1.8	*0.7*
RMSECV [%]	2.2	2.1	*1.8*	2.2	*0.8*

Root mean square error of calibration (RMSEC) and root mean square error of cross validation using leave-one-out method (RMSECV)

**Table 2:** Multiple linear regression equations for the detection of cheese-like product in cheese fat

Wavelength interval (∆*λ*)	Equation
10 nm	% adulterant = −80.3 + (3.8*x*_1_ + 5.3*x*_2_ + 11.3*x*_3_ + 8.5*x*_4_ + 6.3 *x*_5_)/10^5^
30 nm	% adulterant = −117.1 + (6.9*x*_2_ + 7.5*x*_2_ − 2.2*x*_3_ − 4.7*x*_4_ + 4.2*x*_5_)/10^5^
60 nm	% adulterant = −64.6 + (2.2*x*_1_ + 0.6*x*_2_ − 1.3*x*_3_ + 2.5*x*_4_ + 2.8*x*_5_)/10^5^
80 nm	% adulterant = −91.3 + (0.4*x*_1_ + 3.7*x*_2_ + 0.12*x*_3_ − 3.1*x*_4_ + 1.6*x*_5_)/10^5^
Global model	% adulterant = −85.6 + (−0.5*x*_1_ + 1.8*x*_2_ + 1.7*x*_3_ + 0.6*x*_4_ + 3.5*x*_5_)/10^5^

*x*_1_, *x*_2_, *x*_3_, *x*_4_ and *x*_5_—fluorescence intensities obtained at selected wavelength (wavelengths in the order as listed in the section 3.3)

### Detection limits of cheese adulteration with cheese-like products

The regression analysis of synchronous spectra intensities was used to detect limits of adulteration of cheese with cheese-like products for each of the previously selected wavelengths (Table 3). Limits of detection (LOD) were calculated as three times the standard deviation of the intercept and divided by the calibration curve slope. Moreover, multivariate detection limits (MDL), limits for the data obtained at five wavelengths selected by SPA, were calculated by the procedure proposed by Singh (1993). The conditions for the use of this method were fulfilled. The calculated LOD values indicate the advantage of the higher wavelength intervals over the lower ones for adulteration detection. According to the data acquired at the 60- and 80-nm wavelength intervals, the lowest detection limits of adulteration equalled 3.0 and 4.4% at wavelengths 306 and 307 nm, respectively, and were obtained in the region characteristic for tocopherol emission. Calculated MLD were lower than the mean value of LOD calculated for the individual wavelengths. The lowest MLD equalled 3.1% and was obtained at the 60-nm wavelength interval. Very low MLD value (3.4%) was obtained also for the measurements acquired at the 10-nm wavelength interval. This value was significantly lower than the mean value of LOD calculated for the measurements obtained at Δ*λ* = 10 nm (7.1%) as well from the lowest LOD (6.0%).

**Table 3:** Detection limits of adulteration of cheese fat with cheese-like product fat calculated at different wavelength intervals by means of regression analysis

Wavelength interval (∆*λ*)							LOD_min_		LOD_mean_ (%)	MLD (%)
							*λ*_min_ (nm)	LOD (%)		
10 nm	Wavelength (nm)	304	305	311	320	334	304	6.0	7.1	3.4
	LOD (%)	6.0	8.1	7.2	6.1	8.1				
30 nm	Wavelength (nm)	300	317	321	330	344	317	5.7	6.7	4.7
	LOD (%)	6.3	5.7	7.3	6.4	7.7				
60 nm	Wavelength (nm)	300	306	307	312	316	306	3.0	5.4	3.1
	LOD (%)	7.3	3.0	6.4	4.7	5.7				
80 nm	Wavelength (nm)	305	307	310	312	346	307	4.4	5.5	4.0
	LOD (%)	5.5	4.4	5.9	4.8	6.7				

*LOD* limit of detection, *LOD*_*min*_ the lowest limit of detection, *LOD*_*mean*_ mean limit of detection (calculated as an average LOD for five selected wavelengths), *MLD* multivariate detection limit, *λ* wavelength, *λ*_*min*_ the wavelength at which the lowest limit of detection was obtained

The results of the study surpassed the findings obtained with the use of other methods developed to detect milk, butter or cheese adulteration, e.g. traditional fluorescence spectroscopy was able to detect up to 5% of adulteration of butter fat with vegetable oil (Ntakatsane et al. 2013); HPLC enabled the detection of goat milk in sheep cheese with a limit of 5% (Moatsou et al. 2003); reversed-phase HPLC helped to detect cow, sheep and goat admixtures in cheese detected with a limit of 2% (Ferreira and Cacote 2003), whereas the HIC separation of casein isoforms and admixtures of cow, sheep and goat milk and cheese was detected with a limit of 10% (Bramanti et al. 2003).

### Linear discriminant analysis of synchronous fluorescence intensities at selected wavelengths

**Fig. 4:**
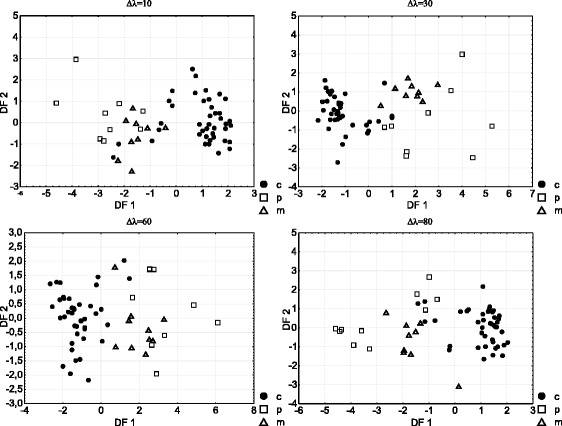
SPA–LDA plots of synchronous fluorescence intensities acquired at different wavelength intervals (*filled circles* indicate cheese fat, *open squares* indicate cheese-like product fat and *open triangles* indicate mixtures of cheese fat with cheese-like product fat)

A linear discriminant analysis was applied to fluorescence intensities of samples of cheese fat and oil from cheese-like products, previously selected by the successive projection algorithm, and to the first five principal components obtained by the use of principal component analysis. PCA was used to reduce the number of dimensions of the data set. The LDA analysis was provided separately for the data acquired at each wavelength interval (10, 30, 60 and 80). The number of classes was set as three (cheese products, cheese-like products and their mixture). The plots of the first two discriminant functions (DF1*DF2) indicate good classification performance of the SPA–LDA analysis (Fig. 4). Figure 4 shows the results of SPA–LDA analysis for classes of cheese, cheese-like product fats and their mixtures in the proportion 1:1 (50% of adulteration). Table 4 presents the percentage of misclassified cheese and cheese-like product fats as well as the total percentage of misclassified samples for cheese and cheese-like product fats. The number of classes for this analysis was set as two (cheese products and cheese-like products). Lower classification errors were obtained for the SPA–LDA rather than for the PCA–LDA method. The lowest classification errors for the PCA–LDA method equalled 3.8% and were obtained for the measurements acquired at the wavelength interval of 10 and 30 nm. The results of fluorescence intensities obtained at the wavelengths interval of 60 nm followed by SPA–LDA analysis enabled the classification of samples without any error.

**Table 4:** Classification errors for SPA–LDA and PCA–LDA in the cheese and cheese-like products fats and mixture data set (%)

SPA–LDA				PCA–LDA			
∆*λ* = 10 nm	∆*λ* = 30 nm	∆*λ* = 60 nm	∆*λ* = 80 nm	∆*λ* = 10 nm	∆*λ* = 30 nm	∆*λ* = 60 nm	∆*λ* = 80 nm
% of misclassified cheese samples							
4.8	0.0	0.0	4.8	4.8	4.8	9.5	2.4
% of misclassified cheese-like product samples							
0.0	20.0	0.0	20.0	0.0	0.0	20.0	20.0
% of misclassified samples (in total)							
3.8	3.8	0.0	7.7	3.8	3.8	11.5	5.8

## Conclusions

The results provided by the experiment have shown that fat contained in cheese and cheese-like products exhibits significant differences in fluorescent intensities. The lowest detection limits (LOD) of adulteration of cheese fat with cheese-like product fat, namely 3.0 and 4.4%, were obtained by the measurements acquired at the 60- and 80-nm wavelength intervals at the excitation wavelengths of 306 and 307 nm. The lowest multivariate limit of detection (MLD) equalled 3.1% and was calculated for the data acquired at the 60-nm wavelength intervals. The MLR models for fluorescence intensities obtained at the wavelength interval of 60 nm enable the prediction of the addition of plant oils with the RMSEC and RMSECV at 1.5 and 1.7, respectively. The RMSEC and RMSECV for the global model equalled 0.7 and 0.8, respectively. It can be concluded that synchronous fluorescence together with the multiple regression technique can be suitable for the quantitative determination of adulteration of hard cheese with cheese-like products.
